# Pharmacokinetics of Continuous Local Antibiotic Perfusion in Fresh-Frozen Cadaver Models of Hip and Knee Arthroplasties

**DOI:** 10.7759/cureus.104181

**Published:** 2026-02-24

**Authors:** Tomomi Mizuhashi, Katsufumi Uchiyama, Koji Sukegawa, Yukie Metoki, Rina Sakai, Daisuke Himeno, Akihiro Maruo, Masashi Takaso, Motoyuki Ogawa, Masanobu Ujihira

**Affiliations:** 1 Graduate School of Medical Sciences, Kitasato University, Sagamihara, JPN; 2 Department of Patient Safety and Healthcare Administration, Kitasato University School of Medicine, Sagamihara, JPN; 3 Department of Orthopedic Surgery, Kitasato University School of Medicine, Sagamihara, JPN; 4 Department of Clinical Anatomy, Research and Development Center for Medical Education, Kitasato University School of Medicine, Sagamihara, JPN; 5 Department of Medical Engineering and Technology, Kitasato University School of Allied Health Sciences, Sagamihara, JPN; 6 Department of Orthopedic Surgery, Chiba-Nishi General Hospital, Matsudo, JPN; 7 Department of Orthopedics and Traumatology, Hyogo Prefectural Harima-Himeji General Medical Center, Himeji, JPN; 8 Department of Anatomy, Kitasato University School of Medicine, Sagamihara, JPN

**Keywords:** continuous local antibiotic perfusion, fresh-frozen cadaver, gentamicin, hip joint, knee joint, periprosthetic joint infection, pharmacokinetics, septic arthritis

## Abstract

Background

Periprosthetic joint infections (PJI) remain a challenging complication of arthroplasty. The aim of using continuous local antibiotic perfusion (CLAP), which consists of intramedullary antibiotic perfusion (iMAP) and negative-pressure intra-articular drainage, is to maintain high local antibiotic concentrations while minimizing systemic exposure. In this study, the anatomical feasibility and pharmacokinetics of CLAP were evaluated in fresh-frozen cadavers.

Methods

A cadaver (female, 90 years old) was used to model total hip arthroplasty (THA), bipolar hemiarthroplasty (BHA), cemented total knee arthroplasty (TKA), and septic native knee. Gentamicin sulfate (1,200 μg/mL) was continuously infused at 2 mL/hour through iMAP pins. In knee models, intra-articular infusion and drainage were performed using a Salem Sump™ tube (Cardinal Health, Dublin, OH). The joint effluent was sampled at 12, 20, 36, and 48 hours and analyzed using particle-enhanced turbidimetric inhibition immunoassay. After the infusion, 20 mL of crystal violet was injected through iMAP pins to visualize the flow pathways.

Results

Gentamicin concentrations increased over time in both hip models, peaking at 36 hours (total hip arthroplasty: 1,170 μg/mL; bipolar hemiarthroplasty: 930 μg/mL). The native knee concentration remained at ≥1,000 μg/mL after 20 hours. In the cemented TKA model, concentrations remained stable (1,098-1,185 μg/mL); crystal violet did not pass from the iMAP system into the joint, indicating that the cement was blocking marrow-joint communication. Dye studies confirmed intra-articular inflow enhanced by trans-acetabular perforations in hip models. In knee models, the dye largely remained within the marrow unless a direct osseous-articular conduit was present.

Conclusions

In this cadaveric study, we demonstrated that the use of CLAP enabled high intra-articular gentamicin levels to be maintained when communication existed between the iMAP system and the joint cavity. Trans-acetabular perforations improved perfusion in the hip, whereas cemented TKA impeded diffusion, indicating that delivery routes must be adapted to the fixation status. Because this cadaveric model is based on anatomical and mechanical assumptions and lacks physiological processes such as blood flow and drug clearance, the observed pharmacokinetics should not be interpreted as directly equivalent to in vivo conditions. Although limited because only a single cadaver was used and living physiology was absent, these findings provide foundational anatomical and pharmacokinetic insights to support the optimization of CLAP in clinical practice.

## Introduction

Total joint arthroplasty is a treatment that reliably relieves pain and restores function; however, periprosthetic joint infections (PJI) remain a challenging complication to treat and prevent. Reported incidence rates of PJI are 0.8%-1.2% after total hip arthroplasty (THA) and 0.9%-1.3% after total knee arthroplasty (TKA) [1]. Patients presenting early and meeting specific criteria may be candidates for debridement, antibiotic medication, or implant retention [2]. In addition to these criteria, implant removal is often recommended. However, removal can be technically demanding or prohibitive in patients who are older, have osteoporosis, or exhibit frailty. Therefore, a strategy that suppresses infection and preserves well-fixed implants is desirable.

In cases of PJI, perfusion to the infected bone and adjacent soft tissue is often impaired, limiting the distribution of intravenously administered antibiotics. Furthermore, bacterial biofilm formed on implant surfaces markedly increases the required therapeutic threshold. The minimum inhibitory concentration represents the lowest antibiotic concentration required to prevent the visible growth of free-floating bacteria, whereas the minimum biofilm eradication concentration represents the concentration necessary to eliminate sessile biofilm-embedded bacteria. As the biofilm matrix provides physical and metabolic barriers, the minimum biofilm eradication concentration is typically 100-1,000 times higher than that of the minimum inhibitory concentration [3-6]. Achieving such systemic concentration without inducing toxicity is virtually impossible. To overcome these limitations, Maruo et al. introduced continuous local antibiotic perfusion (CLAP), a novel strategy designed to maintain high antibiotic concentrations at the infection sites [7]. In CLAP, a concentrated solution of antibiotic is continuously infused at a controlled rate through intramedullary antibiotic perfusion (iMAP) pins, whereas intra-soft tissue antibiotic perfusion (iSAP), which refers to antibiotic perfusion within the periarticular soft tissues facilitated by negative pressure, is concurrently performed using a double-lumen drainage tube. With this configuration, antibiotics can perfuse the infected compartment and be continuously collected via suction, thereby sustaining therapeutic levels that approach the minimum biofilm eradication concentration while minimizing systemic exposure.

CLAP differs fundamentally from conventional suction irrigation systems because the control of dead space under continuous negative pressure and the slow and steady delivery of a high concentration of antibiotics are combined. Preliminary clinical series have demonstrated its feasibility in debridement, antibiotic perfusion, and implant retention procedures for PJI. However, basic anatomical and pharmacokinetic evidence supporting its mechanism of action remains limited, which prompted the present cadaveric investigation.

## Materials and methods

Institutional approval for cadaveric surgical research was obtained before the study. The Kitasato University School of Medicine and Hospital Ethics Committee issued approval B22-177. A single fresh-frozen cadaver (female, 90 years old; height, 138 cm; and weight, 43 kg), donated through an institutional body donation program, was used. Both hips and knees were prepared to simulate representative arthroplasty and native joint conditions. The right hip was subjected to THA using a cementless acetabular component (46 mm E1 liner) and a femoral stem (133°, size 7) with a 28 mm cobalt-chromium head. The left hip underwent a bipolar hemiarthroplasty (BHA) with a CLS® Spotorno® stem (135°, size 6) and a 42 mm outer head (Zimmer Biomet, Warsaw, IN). Cemented cruciate-retaining TKA was performed on the left knee using a femoral component (size C), tibial baseplate (size 3), and polyethylene insert. The right knee was used as the native (nonimplanted) joint model.

To evaluate CLAP, two complementary delivery-drainage systems were employed. The iMAP system utilizes 5 mm pins (Cubex Medical, Tokyo, Japan) inserted to deliver gentamicin directly into the peri-implant bone marrow adjacent to the joint. The intra-articular soft tissue access and drainage system comprised a 20-French Salem Sump™ double-lumen tube (Cardinal Health, Dublin, OH) inserted by entry into the joint; its wider lumen was connected to a negative-pressure wound therapy (NPWT) device (RENASYS TOUCH®, Smith & Nephew plc, London, United Kingdom) that applied continuous suction at -60 mmHg, while the narrower lumen was used for intra-articular infusion when specified.

**Figure 1:**
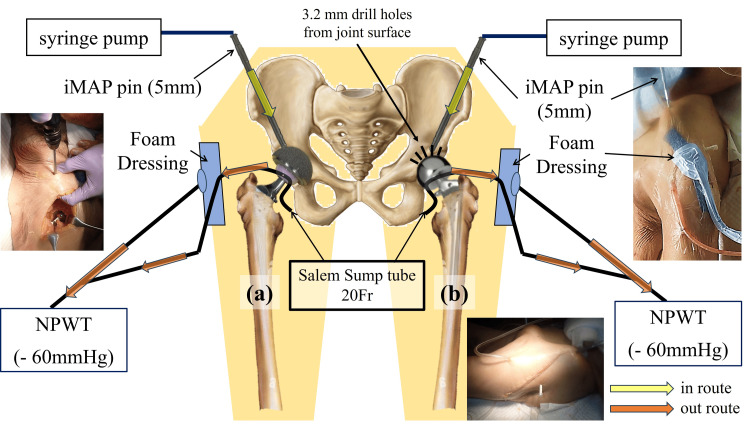
Experimental setup for the hip joint models Schematic diagrams illustrating the experimental setup for intramedullary antibiotic perfusion (iMAP) in the hip joint models. (a) In the total hip arthroplasty (THA) model, a single iMAP pin was inserted from the iliac crest toward the acetabular region to deliver gentamicin into the iliac bone marrow, from which antibiotics could enter the joint cavity through marrow-joint communication. Intra-articular drainage was achieved using a Salem Sump™ tube connected to negative-pressure wound therapy. (b) In the bipolar hemiarthroplasty (BHA) model, the same iMAP and drainage configuration was used, with additional trans-acetabular perforations created to facilitate antibiotic flow from the iliac bone marrow into the joint cavity, thereby enhancing intra-articular perfusion NPWT: negative-pressure wound therapy

For the hip models, THA configuration included one iMAP pin advanced from the iliac crest toward the acetabular cup and Salem Sump™ tube placed intra-articularly with negative pressure drainage (Figure 1a). Communication between the iMAP pin and the joint cavity was confirmed by saline reflux (Figure 1b). In the BHA model, 10 3.2 mm trans-acetabular perforations were drilled from the joint surface into the ilium to facilitate bone-to-joint communication, after which the same iMAP and iSAP setup as in the THA model was established.

**Figure 2:**
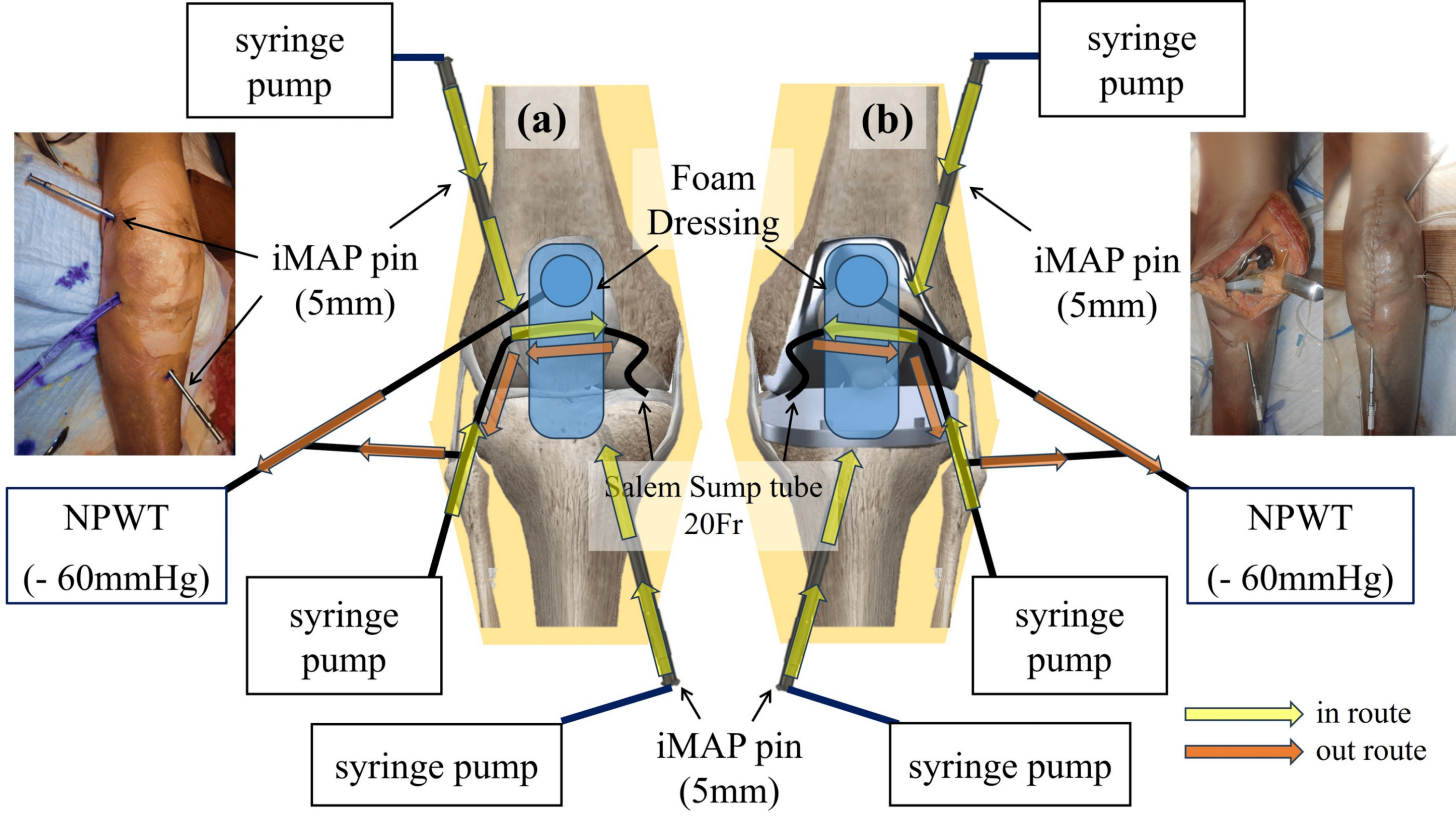
Experimental setup for the knee joint models Schematic diagrams illustrating the experimental setup for intramedullary antibiotic perfusion (iMAP) and intra-soft tissue antibiotic perfusion (iSAP) in the knee joint models. (a) In the native knee model, iMAP pins were placed in the femur and tibia to deliver antibiotics into the bone marrow, while a Salem Sump™ double-lumen tube was inserted intra-articularly to enable direct intra-articular infusion and continuous negative-pressure drainage (iSAP). (b) In the cemented total knee arthroplasty (TKA) model, iMAP pins were positioned adjacent to the cement-bone interface of the femoral and tibial components. Because cement fixation prevents marrow-joint communication, intra-articular antibiotic delivery and drainage were primarily achieved via the Salem Sump™ tube, allowing differentiation between intramedullary and intra-articular perfusion pathways NPWT: negative-pressure wound therapy

For the knee models, both femoral and tibial iMAP pins were placed, with the femoral pin inserted through the lateral supracondylar region and the tibial pin from the anteromedial aspect. The insertion depth was anatomically adjusted to position each pin tip within the cancellous bone of the distal femur and proximal tibia adjacent to the joint line (approximately 30-40 mm) (Figure 2). In the native knee model, the Salem Sump™ tube was inserted into the suprapatellar pouch; its narrow lumen was used for intra-articular infusion and its wide lumen for continuous negative-pressure drainage. No intentional bone-joint perforations were observed in this model (Figure 2a). The TKA model included cemented femoral and tibial components. iMAP pins were placed adjacent to the cement interfaces so that the infused gentamicin solution could reach the underlying bone marrow, and the same intra-articular tube was positioned under the patella. Owing to the cemented fixation, trans-osseous perforations connecting the marrow and joint space could not be made (Figure 2b).

Gentamicin sulfate (1,200 μg/mL) was continuously infused at a rate of 2 mL/hour per iMAP pin using independent syringe pumps. In the knee models, an additional intra-articular infusion through the narrow lumen of the Salem Sump™ tube was performed as described previously. The effluent from the intra-articular tube (iSAP drainage) was collected at 12, 20, 36, and 48 hours. The collected samples were obtained exclusively from the intra-articular lumen of the Salem Sump™ tube directly connected to the joint cavity. Although the NPWT system was also applied over the form dressing, no measurable exudate or fluid output was observed from the dressing site throughout the experimental period. Therefore, the analyzed effluent did not represent a mixture of multiple outflow routes, and dilution by fluid originating from the dressing interface did not occur. The concentration of gentamicin in the effluent was measured using a particle-enhanced turbidimetric inhibition immunoassay (Dimension® EXL™ 200, Siemens Healthineers AG, Erlangen, Germany). In the knee models, an intra-articular injection was administered through the same double-lumen tube used for collecting the drainage fluid. Therefore, the measured concentrations primarily represent the local drainage fluid collected under negative pressure and may not reflect the average synovial fluid concentration in the entire joint compartment.

Dye tracking study

After the completion of the antibiotic infusion, 20 mL of crystal violet was slowly injected via iMAP pins. The hips were dissected, and the acetabulum was split along the iMAP trajectory to visualize the staining of the marrow and joints. Knees were dissected using a horizontal cut from the joint surface (approximately 2 cm) and longitudinal splits of the femur and tibia. The components of the TKA were removed for marrow staining.

## Results

Gentamicin in the intra-articular effluent

**Figure 3:**
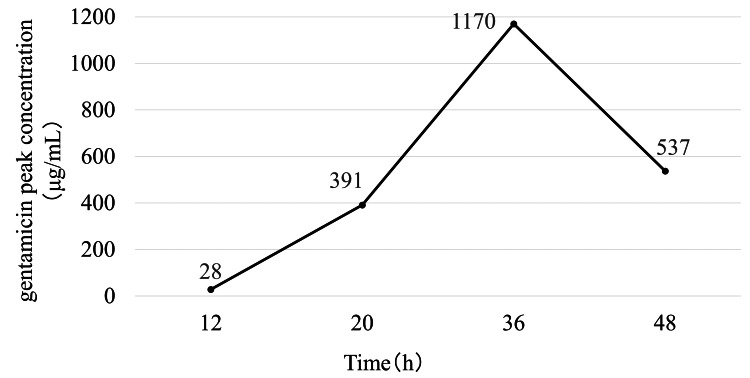
Time-dependent changes in the concentration of gentamicin after right total hip arthroplasty with continuous local antibiotic perfusion The concentration of gentamicin in the hip joint increased progressively and reached a peak at 36 hours; at 48 hours, the concentration had slightly decreased

**Figure 4:**
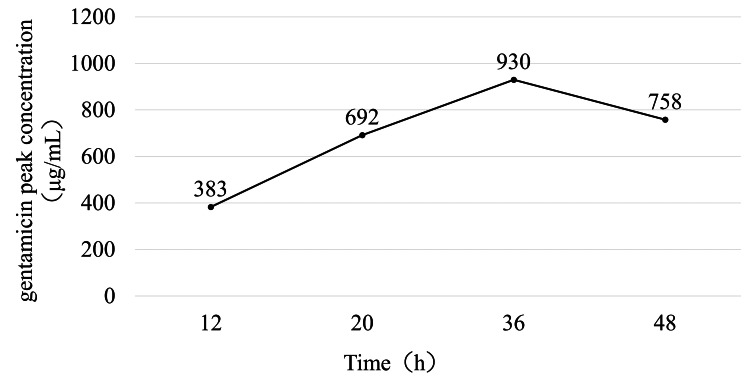
Time-dependent changes in the concentration of gentamicin after the left bipolar hemiarthroplasty with continuous local antibiotic perfusion The concentration of gentamicin in the hip joint increased over time. The concentration decreased after peaking at 36 hours

Pharmacokinetic analysis showed that gentamicin concentration in the intra-articular effluent progressively increased over time in both hip models. In the THA model, the concentration reached 1,170 μg/mL at 36 hours but declined slightly at 48 hours (Figure 3). A similar trend was observed in the BHA model, in which the concentration increased to 930 μg/mL at 36 hours and decreased thereafter (Figure 4).

**Figure 5:**
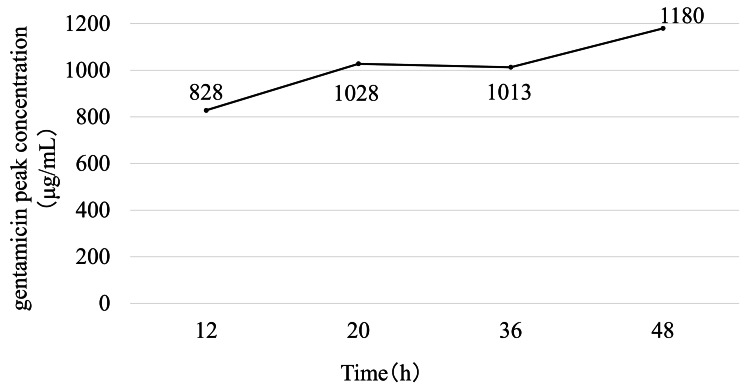
Time-dependent changes in the concentration of gentamicin in the right native knee with continuous local antibiotic perfusion The concentration of gentamicin in the native knee joint remained consistently high throughout the 48-hour perfusion period

**Figure 6:**
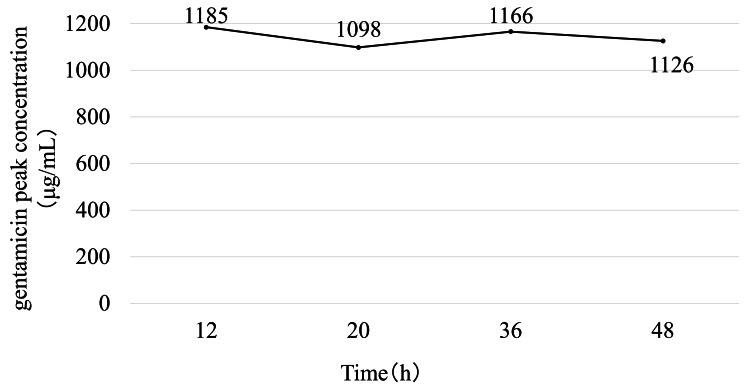
Time-dependent changes in the concentration of gentamicin after left total knee arthroplasty with continuous local antibiotic perfusion The concentration of gentamicin in the cemented knee arthroplasty model remained stable, showing no significant decline and thus indicating limited diffusion due to implant fixation

In the native knee model, the concentration increased rapidly to 1,028 μg/mL at 20 hours and remained consistently above 1,000 μg/mL throughout the 48-hour observation period (range: 1,013-1,180 μg/mL; Figure 5). A stable, high concentration was maintained in the TKA model throughout sampling, ranging from 1,098 μg/mL to 1,185 μg/mL with minimal fluctuation over time (Figure 6).

Crystal violet tracking

**Figure 7:**
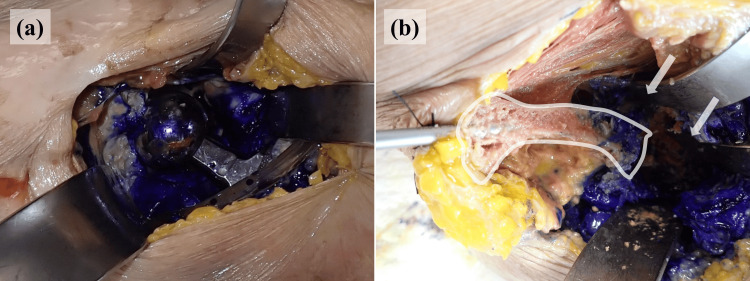
Staining pattern in the right hip joint and the cross-section of the bone The dissection of the right hip joint reveals clear intra-articular staining (a). The area outlined in white indicates the portion of the ilium that was split along the pin track for intramedullary antibiotic perfusion (b). White arrows show stained regions within the joint cavity (b)

**Figure 8:**
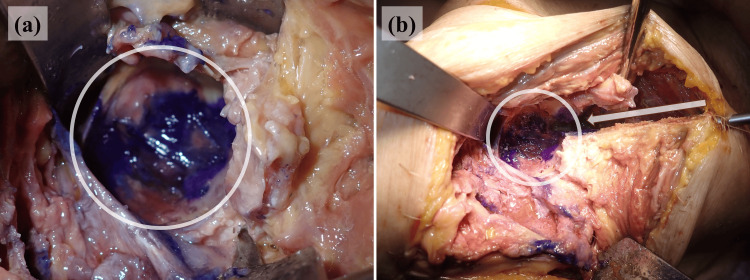
Staining pattern in the left hip joint and the cross-section of the bone In the left hip joint, the dye flows from the tip of the intramedullary antibiotic perfusion pin through the drilled acetabular holes into the joint cavity (a). The white circle delineates the intra-articular region, and the arrow shows the direction in which the dye spreads along the pin (b)

Crystal violet tracking further illustrates the differences in the perfusion pathways among the models. Distinct intra-articular staining was observed in both hips. In the THA model, crystal violet diffused from the tip of the iMAP pin into the joint space, confirming direct communication between the bone marrow and the joint cavity (Figure 7). In the BHA model, the dye readily passed through the intentionally drilled trans-acetabular perforations, and the flow from the iliac bone into the joint was clearly visualized (Figure 8).

**Figure 9:**
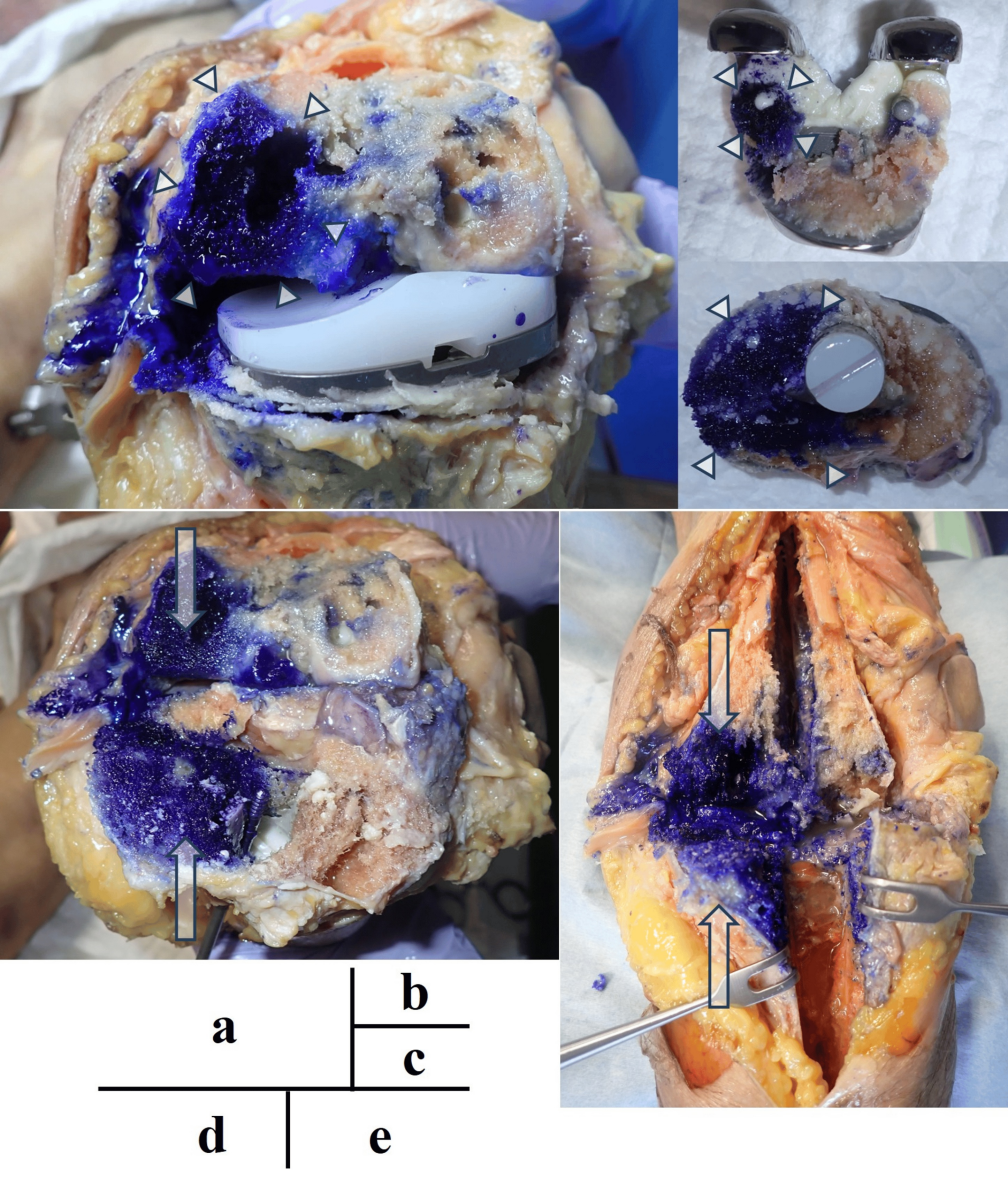
Staining pattern in the left knee joint after removing the artificial components The dye injected through the intramedullary antibiotic perfusion pins is spread along the medial aspects of the distal femur (a, b, d, and e) and proximal tibia (c-e) but has not entered the joint space because of obstruction by the implant. The white arrows and arrowheads indicate the stained areas

**Figure 10:**
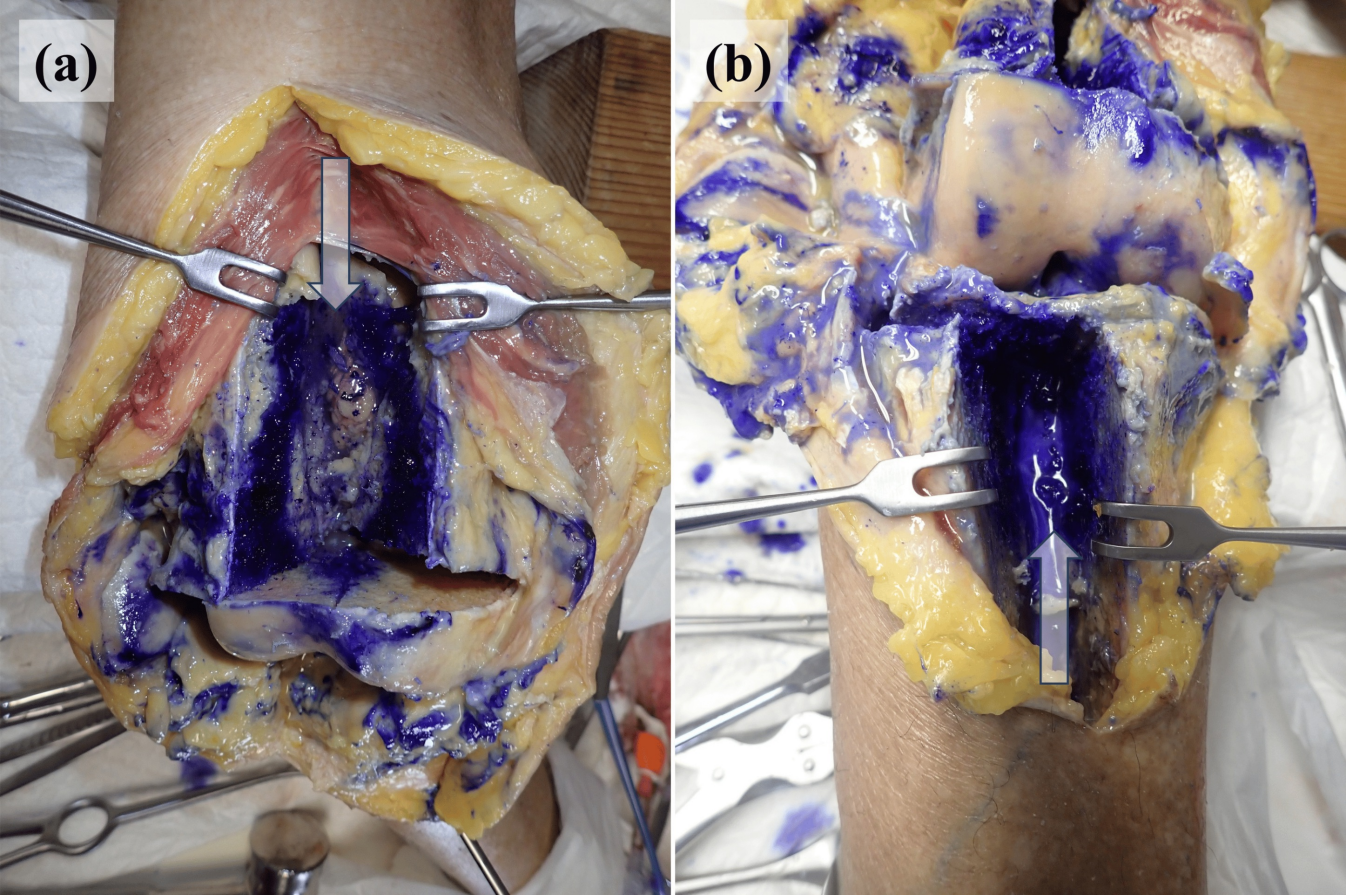
Staining pattern in the right knee joint and the cross-section of the bone Intra-articular staining is observed along the Salem Sump™ tube, while the extensive staining of the bone marrow can be noted in the distal femur and proximal tibia along the pin tracks for intramedullary antibiotic perfusion. No sign of dye entering the joint space is visible

In the knee models, the dye predominantly stained the bone marrow of the distal femur and proximal tibia through iMAP pins but did not penetrate the joint cavity in the cemented TKA model, indicating that the cement barrier prevented passage through the joint (Figure 9). Similarly, in the native knee model without deliberate bone-joint perforations, crystal violet remained confined to the marrow space (Figure 10). In both knee configurations, intra-articular staining was observed through the iSAP double-lumen tube, confirming that the intra-articular drainage route provided direct access to the joint cavity, even in the absence of osseous communication.

## Discussion

In this study, we used a fresh-frozen cadaver model. This model retains soft tissue flexibility and structural integrity that more closely resembles those of living tissues than formalin-fixed cadavers. Thus, fresh-frozen cadavers have been widely adopted for surgical training, anatomical education, and biomechanical research [8-11]. In contrast, formalin fixation hardens and dehydrates tissues, including muscles and connective structures, potentially altering the diffusion and concentration of infused antibiotics. Because near-physiological tissue permeability is preserved in fresh-frozen cadavers, they are considered suitable for simulating intra-articular antibiotic perfusion. Therefore, the use of fresh-frozen cadavers enables a more realistic assessment of local drug kinetics within the joint space.

CLAP has recently emerged as a promising adjunctive treatment for challenging musculoskeletal infections such as open fractures, fracture-related infections, and chronic osteomyelitis [12-14]. Using this method, high concentrations of antibiotics are directly maintained at the infection site while minimizing the systemic exposure and related toxicity [7]. Local negative-pressure drainage promotes perfusion and facilitates the removal of exudates and necrotic debris. Although several clinical studies have reported the efficacy of CLAP in debridement, antibiotic perfusion, and implant retention procedures for PJI, the pharmacokinetic basis of this therapy has not yet been clearly elucidated [15-18]. Thus, the present cadaveric study provides important foundational data on intra-articular antibiotic diffusion under CLAP conditions.

Maruo et al. demonstrated that negative pressure enhances the spread of antibiotics in a cadaver model of the tibia [19]. Consistent with this finding, experiments conducted on the hip showed that negative pressure applied via double-lumen tubes promoted antibiotic diffusion through iMAP pins placed in the ilium into the joint cavity. The measured concentration of antibiotics in the hip joint increased progressively and reached values approximating continuous intravenous infusion levels (approximately 1,200 μg/mL). In both THA and BHA models, similar peak concentrations were achieved within 36 hours, followed by a decline at 48 hours, likely reflecting the leakage of fluids from the tissue and the decomposition inherent to cadaveric specimens. Crystal violet injection further confirmed that the infused dye remained confined to the joint space rather than the bone marrow, simulating the postoperative conditions of THA, in which antibiotics that flow from the iMAP pins can access the joint cavity. However, in vivo, subsequent bone ingrowth around the acetabular cup may restrict such flow, suggesting that intra-articular perfusion from iMAP pins may be effective only during the early postoperative phase. In the BHA model, additional intra-acetabular perforations drilled successfully enhanced perfusion into the joint space, although potential cartilage damage remains a clinical concern when considering similar approaches.

In the knee models, antibiotics were infused through iMAP pins inserted into the femur and tibia, while intra-articular infusion was performed through Salem Sump™ (iSAP) tubes, analogous to the system used in the clinical management of septic arthritis. The rapid increase in intra-articular antibiotic concentrations was primarily attributable to direct infusion via the iSAP tube. In contrast, in the cemented TKA model, the fixation of the components with bone cement likely obstructed intramedullary flow from the iMAP pins. Crystal violet staining confirmed that in the absence of an osseous-articular communication pathway, dye injected through the iMAP pins did not migrate into the joint cavity but remained confined within the medullary canal. These findings suggest that antibiotics delivered via iMAP may not be retrieved through the intra-articular drainage system when no direct marrow-joint communication exists. Although systemic circulation was absent in this cadaveric model, in vivo, such retained antibiotics could potentially enter the bloodstream through the intraosseous vascular network. Therefore, unless intra-articular perforations or communication pathways are established, antibiotics infused via iMAP pins may not effectively reach the joint cavity, and excessive intramedullary concentrations may theoretically increase systemic exposure, emphasizing the importance of the careful consideration of perfusion pathways and the monitoring of antibiotic dosing [19]. In the cemented TKA model, crystal violet injected into the medullary canal did not demonstrate lateral migration. This limited transverse spread may be explained by the trabecular microarchitecture of cancellous bone, which creates compartmentalized spaces that restrict diffusion under static, non-physiological conditions. Furthermore, the surrounding bone cement may have acted as a mechanical barrier, limiting pressure-driven redistribution within the marrow cavity. In the absence of vascular perfusion in this cadaveric model, there was no physiological flow to promote lateral dissemination.

In the early phase of knee PJI, when the infection is confined to the joint, maintaining a high local concentration through iSAP tubes may suffice. In late-phase PJI, when the infection extends into the bone and the implant remains stable, a combined treatment consisting of placing iMAP pins in the infected bone and providing a communication pathway to the joint cavity may enhance local delivery. This approach suppresses infection while the implant is retained, offering a less invasive alternative for medically fragile patients [15,16,20].

This study has a few limitations. First, only a single cadaver specimen was analyzed, precluding generalization and statistical interpretation. Second, a cadaveric model cannot replicate living physiological conditions, such as blood flow, immune response, or tissue regeneration; these factors may affect antibiotic diffusion and absorption, particularly near the bone-implant interface. Third, the progressive decomposition of fresh-frozen cadaver may alter the tissue fluid content over time, influencing the measured concentrations and preventing long-term observation.

In the knee models, the concentration measured from the iSAP drainage was used as a surrogate for intra-articular concentration. However, because the collection port opens immediately within the joint cavity, the sampled fluid likely contained directly infused antibiotics rather than equilibrated joint fluid. Consequently, the measured concentration may have overestimated the actual intra-articular levels.

In vivo, a proportion of locally administered high-concentration antibiotics would inevitably enter the systemic circulation through intraosseous and periarticular vascular networks, contributing to systemic distribution and drug clearance. In clinical settings, additional factors such as hematoma formation and joint effusion may further influence both clearance and intra-articular dispersion. However, because this cadaveric model lacks physiological blood flow, vascular absorption, and renal elimination, systemic absorption and pharmacokinetic clearance could not be assessed. Moreover, both clearance and dynamic dispersion are minimal under cadaveric conditions. Therefore, the measured concentrations primarily reflect local retention and mechanically driven distribution under non-physiological conditions rather than the complete pharmacokinetic behavior observed in living patients.

Despite these limitations, our findings provide valuable mechanistic insights into the pharmacokinetics of CLAP in native and prosthetic joint infections. In particular, the findings reveal how iMAP and iSAP systems influence antibiotic distribution within the joint. Future translational research should include the in vivo quantification of antibiotic concentrations in patients undergoing CLAP and correlating these with clinical efficacy and safety outcomes. Such data are essential for refining CLAP protocols and supporting their standardized application in the management of orthopedic infections.

## Conclusions

This cadaveric simulation demonstrated the pharmacokinetic characteristics of CLAP in both native and prosthetic joints. Using a fresh-frozen cadaver model that closely mimicked living tissue, sustained high local concentrations of antibiotics were achieved when iMAP pins and intra-articular Salem Sump™ (iSAP) tubes were properly positioned. In the hip model, drilling intra-acetabular perforations markedly enhanced intra-articular perfusion, whereas implant fixation limited the perfusion pathways in the knee model, confirming the need for alternative or modified infusion routes. Although this study was limited by the use of a single specimen and the lack of a physiological response, the results confirm the anatomical and procedural feasibility of CLAP and provide a foundation for optimizing clinical protocols.
